# How often is occult atrial fibrillation in cryptogenic stroke causal vs. incidental? A meta-analysis

**DOI:** 10.3389/fneur.2023.1103664

**Published:** 2023-03-14

**Authors:** Napasri Chaisinanunkul, Shaan Khurshid, Brian H. Buck, Alejandro A. Rabinstein, Christopher D. Anderson, Michael D. Hill, Jennifer E. Fugate, Jeffrey L. Saver

**Affiliations:** ^1^Department of Neurology, Phyathai 1 Hospital, Bangkok, Thailand; ^2^Demoulas Center for Cardiac Arrhythmias and Cardiovascular Research Center, Massachusetts General Hospital, Boston, MA, United States; ^3^Division of Neurology, University of Alberta, Edmonton, AB, Canada; ^4^Department of Neurology, Mayo Clinic, Rochester, MN, United States; ^5^Department of Neurology, Brigham and Women's Hospital, Boston, MA, United States; ^6^Department of Clinical Neuroscience and Hotchkiss Brain Institute, University of Calgary, Calgary, AB, Canada; ^7^Department of Neurology, University of California, Los Angeles, Los Angeles, CA, United States

**Keywords:** cryptogenic stroke, atrial fibrillation, cardiac monitoring, attributable risk, diagnosis, epidemiology

## Abstract

**Introduction:**

Long-term cardiac monitoring studies have unveiled low-burden, occult atrial fibrillation (AF) in some patients with otherwise cryptogenic stroke (CS), but occult AF is also found in some individuals without a stroke history and in patients with stroke of a known cause (KS). Clinical management would be aided by estimates of how often occult AF in a patient with CS is causal vs. incidental.

**Methods:**

Through a systematic search, we identified all case–control and cohort studies applying identical long-term monitoring techniques to both patients with CS and KS. We performed a random-effects meta-analysis across these studies to determine the best estimate of the differential frequency of occult AF in CS and KS among all patients and across age subgroups. We then applied Bayes' theorem to determine the probability that occult AF is causal or incidental.

**Results:**

The systematic search identified three case–control and cohort studies enrolling 560 patients (315 CS, 245 KS). Methods of long-term monitoring were implantable loop recorder in 31.0%, extended external monitoring in 67.9%, and both in 1.2%. Crude cumulative rates of AF detection were CS 47/315 (14.9%) vs. KS 23/246 (9.3%). In the formal meta-analysis, the summary odds ratio for occult AF in CS vs. KS in all patients was 1.80 (95% CI, 1.05–3.07), *p* = 0.03. With the application of Bayes' theorem, the corresponding probabilities indicated that, when present, occult AF in patients with CS is causal in 41.2% (95% CI, 15.5–77.7%) of patients. Analyses stratified by age suggested that detected occult AF in patients with CS was causal in 62.3% (95 CI, 0–87.1%) of patients under the age of 65 years and 28.5% (95 CI, 0–63.7%) of patients aged 65 years and older but estimates had limited precision.

**Conclusion:**

Current evidence is preliminary, but it indicates that in cryptogenic stroke when occult AF is found, it is causal in about 41.2% of patients. These findings suggest that anticoagulation therapy may be beneficial to prevent recurrent stroke in a substantial proportion of patients with CS found to have occult AF.

## Introduction

As optimal secondary prevention therapy to prevent recurrent ischemic stroke is determined by the causative mechanism of the index stroke, the diagnosis of the ischemic stroke mechanism is a key element of patient care. Cryptogenic ischemic strokes are symptomatic cerebral infarcts for which no probable cause is identified after standard diagnostic evaluation (1). In general, the percentage of ischemic strokes that are classified as cryptogenic has declined over time, from 40% in the 1970s to 10–20% at advanced centers performing extensive testing, in part due to the recognition of paradoxical embolism through patent foramen ovale as a well-established and not uncommon cause of ischemic stroke (1–3). However, the size of the residual population of patients with cryptogenic stroke remains substantial, and increasing the proportion in whom a specific stroke mechanism can be identified remains an urgent challenge.

Occult, low-burden, atrial fibrillation (AF) is a potential cause of otherwise cryptogenic ischemic stroke. Overt AF is the most common cause of cardioembolic stroke and is present in 2% of people younger than 65 years and 9% in people older than 65 years (4). Anticoagulation is effective for stroke prevention in patients with overt AF and additional vascular risk factors. Long-term cardiac monitoring studies have unveiled low-burden, occult AF in some patients with otherwise cryptogenic stroke (CS) (5). Occult AF is also found in some individuals without a stroke history and in patients with a stroke of a known cause (KS). Clinical management may be aided by estimates of how often occult AF in a patient with CS is causal vs. incidental.

The objective of this review was to provide an estimate of the frequency with which detected occult AF in a patient with CS is causal.

## Materials and methods

We performed a systematic, study-level meta-analysis to aggregate data from studies assessing the frequency of occult atrial fibrillation (AF) in both patients with cryptogenic ischemic stroke or transient ischemic attack (TIA) and patients with a known-cause stroke or transient ischemic attack. The study was performed according to PRISMA guidelines. The systematic search of Medline was performed for the time period between 1 January 2000 and 28 January 2023 using the following search strategy: (transient ischemic attack OR stroke) AND (cryptogenic OR undetermined) AND (atrial fibrillation OR paroxysmal atrial OR occult) AND (continuous monitoring OR Holter OR MOCT OR Ziopatch OR internal loop recorder OR ILR OR pacemaker). Studies were included if two reviewers (NC and JLS) determined that they met the following criteria: (1) a case–control or cohort study with both patients with cryptogenic ischemic stroke or TIA and patients with known-cause ischemic stroke or TIA and (2) prolonged (≥1 week) continuous ambulatory cardiac monitoring for occult AF, using external or internal recorders.

### Data extraction

Full texts of eligible studies were reviewed, and data were directly extracted into electronic data tables. For each study, we extracted journal, publication year, patient age, number of cryptogenic stroke cases and known stroke controls, frequencies of occult AF in both groups, and modality used to monitor for occult AF (external ambulatory monitor and internal loop recorder). Data abstraction was performed independently by two investigators (NC and JLS), and differences were resolved by consensus. In addition, for each of the analyzed studies, salient data items not presented in published reports were provided by co-authors who were original study investigators.

### Meta-analyses of case–control and cohort studies

Random-effects meta-analyses were performed to estimate the summary odds ratio (OR) and 95% CIs of having occult AF in patients with CS vs. patients with KS. Heterogeneity between studies was assessed using Cochran's Q statistics (considered significant at *P* < 0.10) and the I^2^ statistics. All meta-analyses were performed in RevMan 5.3. A subgroup analysis was performed of studies reporting data separately for patients younger than 65 years old and patients who are 65 years and older. Two independent raters (NC and JLS) assessed the risk of bias in cohort studies using the Risk Of Bias In Non-randomized Studies of Interventions (ROBINS-I) cohort study tool in case–control studies, with differences resolved by consensus discussion (5). Data were displayed as forest plots. This review was not pre-registered with the publication of a formal protocol.

### Probability of causal vs. incidental relationship

The frequency with which discovered occult AF is causal or incidental in a patient with CS was calculated using Bayes' theorem, following the method of Alsheikh-Ali et al. (6). The conceptual framework employs two assumptions: (1) If not for those strokes attributable to occult AF, the prevalence of occult AF would be similar in patients with CS compared with KS and (2) CS in patients without detected occult AF is not caused by undetected occult AF. These assumptions permit the calculation of the probability that detected occult AF is incidental or causal based on the prevalence of occult AF in CS cases and in control subjects according to the following equation:


$$\begin{array}{l} Probability\;occult\;AF\;is\;incidental\;in\;CS\;cases=\\ \left[(Prevalence\;of\;occult\;AF\;in\;controls\right)\\ \times (1-Prevalence\;of\;occult\;AF\;in\;CS\;cases)]\\ ÷[(Prevalence\;of\;occult\;AF\;in\;CS\;cases)\\ \times (1-Prevalence\;of\;occult\;AF\;in\;controls)] \end{array}$$

### Statistics

Using the Bayes' theorem-derived equation, we calculated the probability that discovering occult AF in a patient with CS is incidental for each individual study. A summary probability across all studies was calculated with confidence limits based on the natural logarithm of each study's probability that occult AF is incidental and also its weight in a random-effects model.

## Results

**Figure 1:**
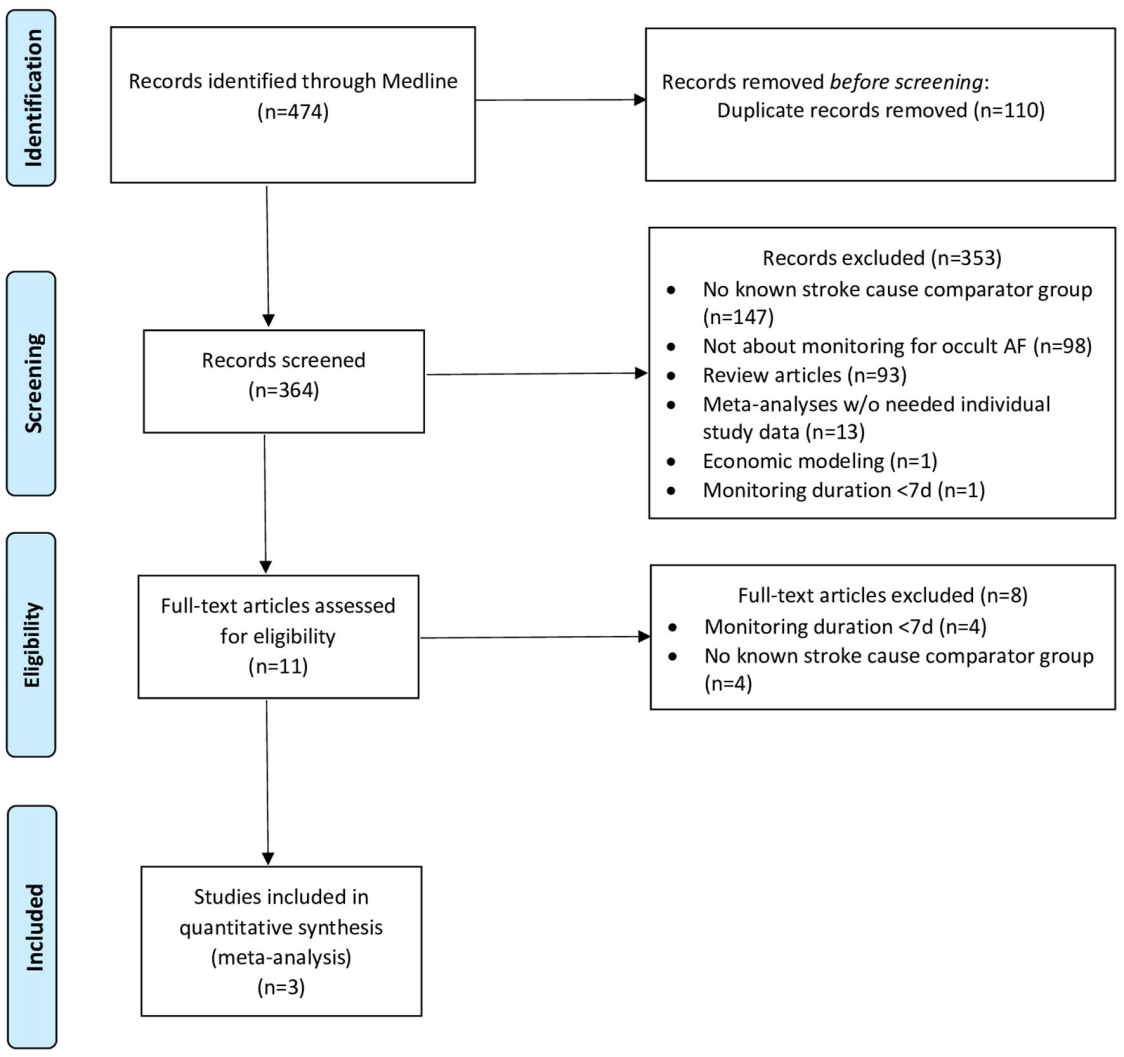
PRISMA flow diagram showing study selection.

The systematic search yielded 364 records for screening, among which 353 were excluded based on study titles and abstracts, and further eight studies were excluded based on the full-text review, yielding three studies meeting selection criteria (Figure 1, Supplementary Table 1) (7–9). The studies included one case–control and two cohort investigations and collectively enrolled a total of 561 patients, including 315 patients with cryptogenic stroke and 246 patients with known-cause stroke. The risk of bias was assessed as low for two studies (one cohort and one case–control) and intermediate for one study (cohort study) (Supplementary Table 2).

Study and patient characteristics are shown in Table 1. Across all studies, the mean age of patients with CS was 65.4 years and that of patients with KS was 64.3 years, and women accounted for 40.6% of patients with CS and 40.2% of patients with KS. The frequencies of subtypes of known stroke mechanisms varied across studies with available data, including large artery atherosclerosis in 24–48%, small vessel disease in 36–45%, and non-AF cardioembolism in 18–39%. Windows for the start of monitoring varied across studies and included up to 3, 6, and 12 months post-stroke. The duration of monitoring also varied. In the two studies providing detailed information, monitoring duration was 3–4 weeks in 228 patients and 12 months in 150 patients. Across all studies, monitoring types were as follows: in CS, external monitoring only in 63.5%, internal monitoring only in 34.9%, and both in 1.9%, and in KS, external monitoring only in 73.6%, internal monitoring only in 26.0%, and both in 0.4%. In the two studies providing details, the durations of AF required to declare AF presents were 30 s and 2 min.

**Table 1:** Characteristics of studies and patients.

	**Rabinstein et al. (7)**	**Khurshid et al. (8)**	**Buck et al. (9)**
Type of study (case-control, cohort, other)	Case control	Cohort	Cohort
Location of study	United States	United States	Canada
Centers, *N*	1	1	3
CS, KS patients, *N, N*	64, 64	74, 75	177, 107
**Age, mean (**±**SD)**			
CS patients	67.9 (±11.0)	65.7 (±15.2)	63.7 (12.5)
KS patients	64.5 (±10.5)	63.4 (±14.8)	64.9 (12.8)
**Sex, female, No (%)**			
CS patients	22 (34.4%)	31 (41.9%)	75 (42.4%)
KS patients	27 (42.2%)	29 (38.7%)	43 (40.1%)
**Stroke mechanism in KS pts, No (%)**			
Large artery atherothrombotic	28 (43.8%)	–	26 (24.3%)
Small vessel	23 (35.9%)	–	48 (44.9%)
Cardioembolic	13 (20.3%)	11 (14.7%)	19 (17.8%)
Other	–	–	8 (7.5%)
2 or more causes	–	–	6 (5.6%)
Any of LAA, SV, Other	–	64 (85.3%)	–
**CHADS Scoring Information**			
CHADS2 ≥ 2, No (%)	CS 29 (45.3%) / KS 31 (48.4%)	–	–
CHA2DS2-VASc score, median (IQR)	–	–	CS 4 (3–5) /KS 4 (3–5)
Start of monitoring	Within 3 m post-stroke	Any time between acute discharge and 12 m	Within 6 m post-stroke
Duration of monitoring	3w	Not specified	ILR pts−12 m (*n* = 150); External pts−4 w (*n* = 150)
**Type of monitor**			
External (Event/Holter) only	CS 100%/KS 100%	CS 60.8%/KS 84.0%	CS 51.5%/KS 50.5%
ILR only	–	CS 31.1%/KS 14.7%	CS 49.4%/KS 49.5%
Both	–	CS 8.1%/KS 1.3%	–
Min AF duration to declare AF present	30 s	Not specified	>2 min
**Frequency of occult AF, No (%)**			
CS patients	16 (25.0%)	10 (13.5%)	21 (11.9%)
KS patients	9 (14.1%)	5 (6.7%)	9 (8.4%)

**Figure 2:**
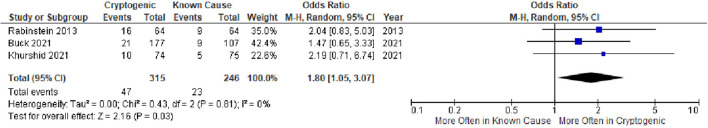
Forest plot of studies comparing frequencies of occult atrial fibrillation in patients with cryptogenic ischemic stroke and patients with ischemic stroke of a known cause. CI, confidence interval.

Rates of the detection of occult AF are shown in Table 1, Figure 1. Crude cumulative rates of AF detection were CS 47/315 (14.9%) vs. KS 23/246 (9.3%). With formal meta-analysis aggregating data across all patients, the odds ratio for detection of occult AF among patients with CS vs. patients with KS was 1.80 (95% CI, 1.05–3.07), *p* = 0.03 (Figure 2). There was no major evidence of heterogeneity across studies, with I^2^ = 0%.

**Figure 3:**
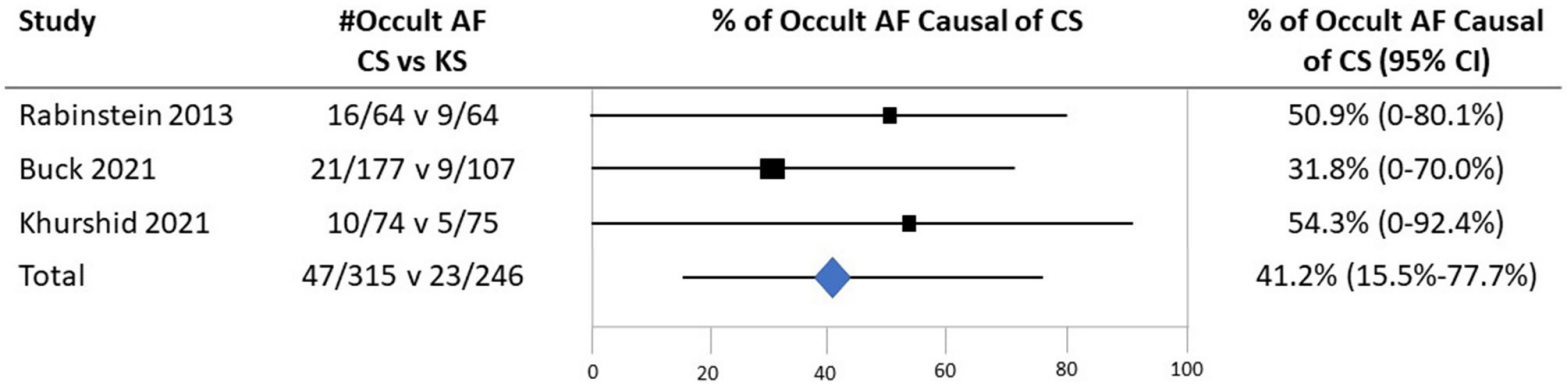
The probability that detected occult atrial fibrillation is causal in patients with CS based on case–control studies examining the prevalence of occult AF in cases with CS vs. controls with the stroke of a determined cause. Individual studies were identified by the first author and year of publication. The second column shows the prevalence of occult AF (# occult AF/total number of patients) in cases vs. controls. Black boxes with sizes corresponding to each study's weight in the analysis represent the point estimate of the probability that the PFO is causal with 95% CIs represented with the gray lines. The diamond in the last row represents the summary estimate of the probability.

With the application of Bayes' theorem, the probabilities that occult AF is causal of cryptogenic stroke are shown in Figure 3. The summary estimate derived from all three studies indicated that when present among patients with cryptogenic stroke, occult AF is causal in 41.2% (95% CI, 15.5–77.7%).

Combining the rate of occult AF presence in patients with CS across all series (14.9%) and the frequency with which the detected occult AF is statistically deemed causal (41.2%) projects that occult AF accounts for 6.1% of all cryptogenic strokes (attributable risk).

**Figure 4:**
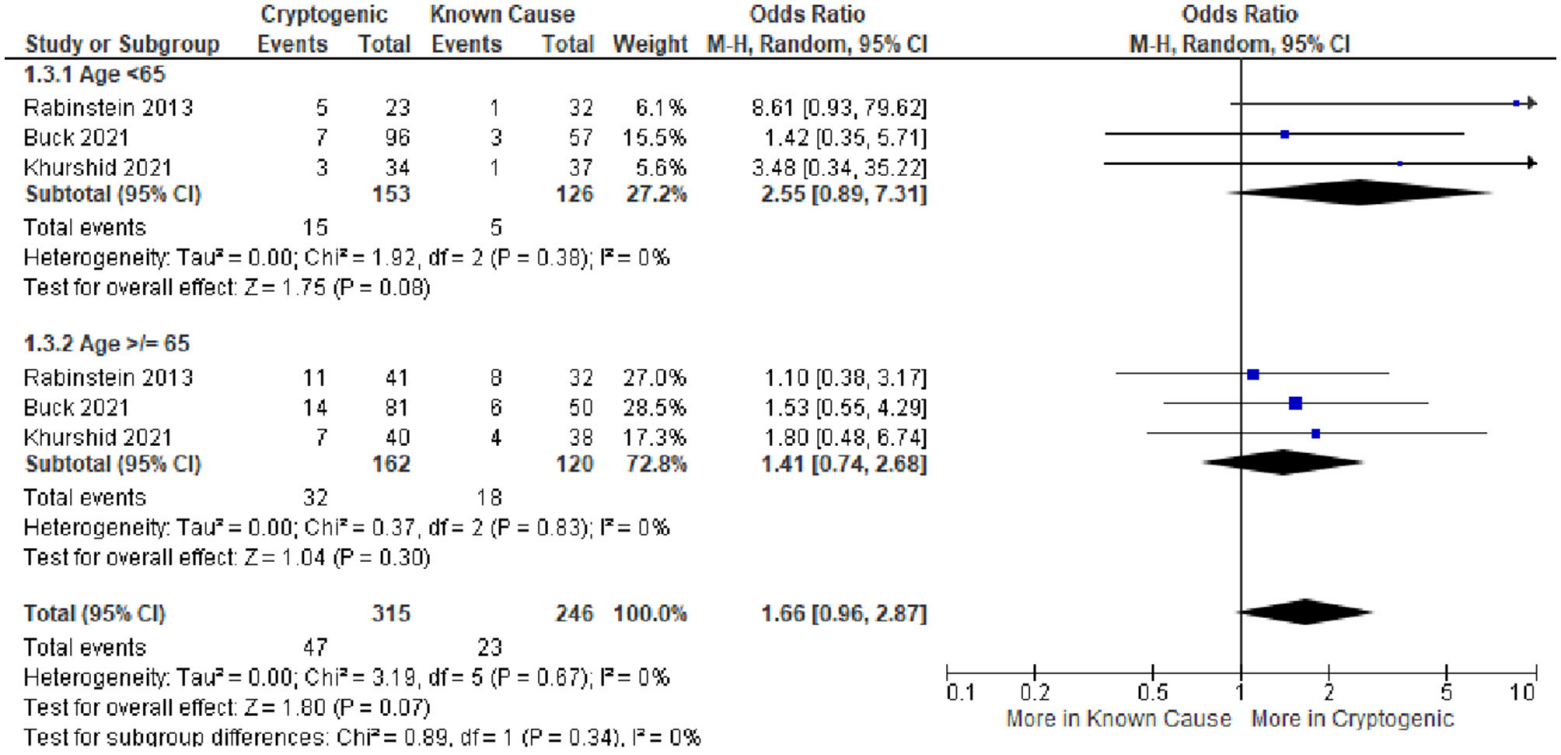
Forest plot of studies comparing frequencies of occult atrial fibrillation in patients with cryptogenic ischemic stroke and patients with ischemic stroke of known cause in the subgroups of people under the age of 65 years and who are of age 65 years and higher. CI, confidence interval.

**Figure 5:**
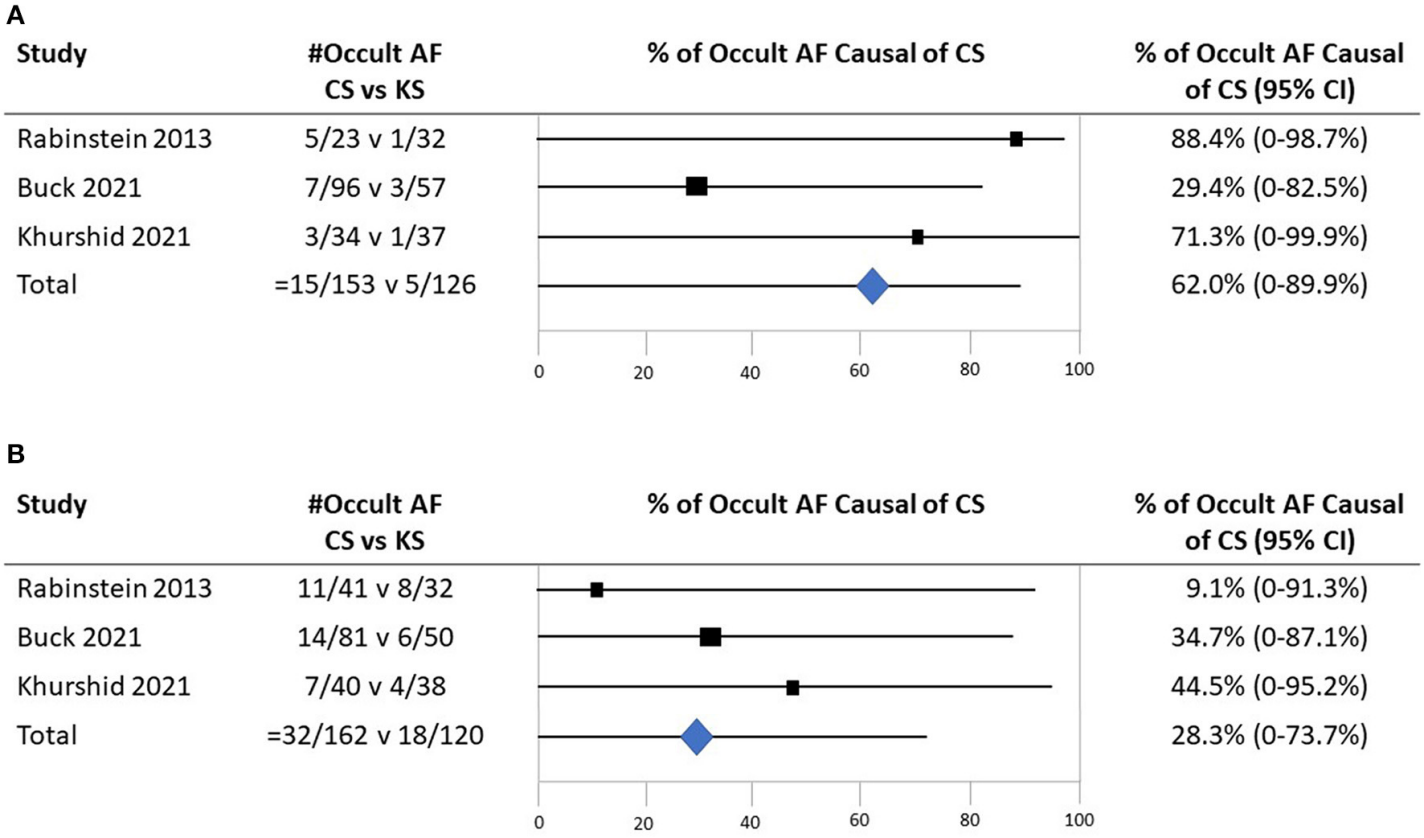
The probability that detected occult atrial fibrillation is causal in patients with CS. **(A)** Among patients under the age of 65 years. **(B)** Among patients 65 years old and higher.

All three studies provided age-specific subgroup data for age stratified at < 65 vs. ≥65 years. In Buck et al.'s study, among 284 patients (177 CS and 107 KS) under the age of 65 years, the mean age was 54.8 (±8.3) years. In Rabinstein et al.'s study, among 55 patients under 65 years (23 CS and 32 KS), the mean age was 55.7 (±6.0) years. In Khurshid et al.'s study, among patients under 65 years (34 CS and 37 KS), the mean age was 51.8 (±9.6) years. Among patients under the age of 65 years, crude cumulative rates of AF detection were CS 15/153 (9.8%) vs. KS 5/126 (4.0%); the odds ratio for detection of occult AF among patients with CS vs. patients with KS was 2.55 (95% CI, 0.89–7.31). Among patients ≥65 years, crude cumulative rates of AF detection were CS 32/162 (19.8%) vs. KS 18/120 (15.0%); the odds ratio for detection of occult AF among patients with CS vs. patients with KS was 1.41 (95% CI, 0.74–2.68) (Figure 4). Applying Bayes' theorem, the frequencies with which discovered occult AF was the cause of stroke in patients with CS patients were as follows: among patients < 65 years, the frequency was 62.3% (95 CI, 0–87.1%), and among patients ≥65 years, it was 28.5% (95 CI, 0–63.7%) (Figure 5).

Combining the rate of occult AF presence in younger patients with CS across all three series (9.8%) and the frequency with which the detected occult AF is statistically deemed causal (62.3%) projects that occult AF accounts for 6.1% of all cryptogenic strokes (attributable risk) in patients under the age of 65 years. Similarly, for older patients, the rate of detection (19.8%) and attributed causality (28.5%) suggest that occult AF accounts for 5.6% (attributable risk) in patients aged 65 years and older.

## Discussion

In this study, Bayesian analysis was employed to estimate the probability that occult atrial fibrillation in a patient with cryptogenic stroke is causally related rather than an incidental finding based on meta-analytically summarized effects from three case–control and cohort studies. Findings indicated that when identified in patients with CS, occult AF is causally related to the index stroke in 41% and incidental in 59%. This relationship varied with patient age: among patients under 65 years old, detected occult AF is causally related in nearly two-thirds of patients, while among patients who are 65 years and older, detected occult AF is causally related in more than one-quarter of patients. Given the frequency with which occult AF is detected among patients with CS, the results suggest that occult AF is an important mechanism of CS, accounting for 1 in 16 of all cases of CS, including 1 in 16 cases in patients under the age of 65 years, and 1 in 18 cases in patients who are 65 years and older.

These findings are perforce consistent with prior studies as they are based on the meta-analytic aggregation of prior findings. The results are constrained by the somewhat surprising paucity of the case–control and cohort–control studies on occult atrial fibrillation in the literature. Whereas a similar investigation of patent foramen ovale as a cause of cryptogenic stroke identified 23 studies for meta-analytic combination (6), the current investigation identified only three studies on occult AF. These findings suggest that more and larger case–control studies on occult AF in cryptogenic vs. known-cause patients with stroke are an important priority for further investigation.

The data in this study suggesting that occult AF is less frequently the cause of index stroke in older than in younger patients are consistent with the known increase in the prevalence of mildly stenotic atherosclerosis as a competing source in elderly populations (10). It is important to note that as more strokes, in general, occur in individuals over the age of 65 years, occult AF would account for an increasing absolute number of events in older patients, despite a reduced attributable fraction.

It is instructive to compare the findings in this study for a causal relationship between occult AF to CS in young and middle-aged patients and those found in the studies on patent foramen ovale (PFO), which is another known source for otherwise cryptogenic stroke (6, 11). Only an indirect comparison can be made as the data available for PFO causal relationship studies define young and middle-aged patients as under the age of 55 vs. under the age of 65 years for the occult AF causal relationship studies. In the PFO studies with adjustment for publication bias, among individuals under the age of 55 years, PFOs were identified in 51% of patients and, per Bayes' theorem, were causally related in 75% of these patients (11). Accordingly, PFO was causally related to 38% of all cryptogenic strokes in young and middle-aged patients. In the occult AF studies, among individuals under the age of 65 years, occult AF was identified in 10% of patients and, per Bayes' theorem, was causally related in 62% of these patients. Consequently, occult AF was causally related to 6% of all CS in young and middle-aged patients. These findings suggest that occult AF is an important cause of CS in young and middle-aged patients with stroke, though not as frequent as PFO. Together, PFO and occult AF account for nearly half (44%) of young to middle-aged cryptogenic strokes. The identification of these mechanisms has substantially reduced the proportion of patients in whom a causal source for an ischemic stroke cannot be identified.

This study has limitations. First, the number of identified case–control and cohort studies and the patients they enrolled was modest, causing wide confidence intervals around all estimates. Accordingly, all study findings should be taken as preliminary. More comparative case–control studies and cohort studies on occult AF in known-cause vs. cryptogenic ischemic stroke are highly desirable. Second, two of the three studies analyzed were cohort studies, with the inherent limitations of selection bias and possible differential intensity in the investigation for occult AF in known-cause vs. cryptogenic stroke. Third, different studies employed different starting times to look for AF, different monitoring durations for the detection of AF, and different modalities (external and internal recording) for the detection of AF. These differences reflect the range of current practice and may reduce the precision of findings. Our use of random-effects, rather than fixed-effects, meta-analysis mitigates but does not completely control this aspect. Fourth, the initiation of long-term monitoring was not started immediately upon discharge in many patients and may have missed early episodes of AF.

In conclusion, the preliminary findings from this study indicate that, when detected, occult AF is likely to be causal in nearly two-thirds of young to middle-aged patients with cryptogenic ischemic stroke and more than one-quarter of older patients with cryptogenic ischemic stroke. These findings suggest that anticoagulation therapy may be beneficial to prevent recurrent stroke in a substantial proportion of patients with CS found to have occult AF.

## Data availability statement

The original contributions presented in the study are included in the article/Supplementary material, further inquiries can be directed to the corresponding author.

## Author contributions

NC and JS contributed to the conception and design of the study and performed the statistical analysis. SK, BB, AR, CA, MH, and JF contributed data. NC organized the database and wrote the first draft of the manuscript. JS wrote sections of the manuscript. All authors contributed to the manuscript revision, read, and approved the submitted version.
