# Corrigendum: How often is occult atrial fibrillation in cryptogenic stroke causal vs. incidental? A meta-analysis

**DOI:** 10.3389/fneur.2023.1206563

**Published:** 2023-05-10

**Authors:** Napasri Chaisinanunkul, Shaan Khurshid, Brian H. Buck, Alejandro A. Rabinstein, Christopher D. Anderson, Michael D. Hill, Jennifer E. Fugate, Jeffrey L. Saver

**Affiliations:** ^1^Department of Neurology, Phyathai 1 Hospital, Bangkok, Thailand; ^2^Demoulas Center for Cardiac Arrhythmias and Cardiovascular Research Center, Massachusetts General Hospital, Boston, MA, United States; ^3^Division of Neurology, University of Alberta, Edmonton, AB, Canada; ^4^Department of Neurology, Mayo Clinic, Rochester, MN, United States; ^5^Department of Neurology, Brigham and Women's Hospital, Boston, MA, United States; ^6^Department of Clinical Neuroscience and Hotchkiss Brain Institute, University of Calgary, Calgary, AB, Canada; ^7^Department of Neurology, University of California, Los Angeles, Los Angeles, CA, United States

**Keywords:** cryptogenic stroke, atrial fibrillation, cardiac monitoring, attributable risk, diagnosis, epidemiology

In the published article, there was an error in Figure 3 as published. For the study in the 2^nd^ row and Totals in the 4^th^ row of the figure, the numerator values, denominator values, point estimates, and confidence intervals were not correct. The corrected Figure and its caption appear below.

In the published article, there were errors in those text passages that stated data values based on Figure 3. The following corrections have been made:

**Figure 3:**
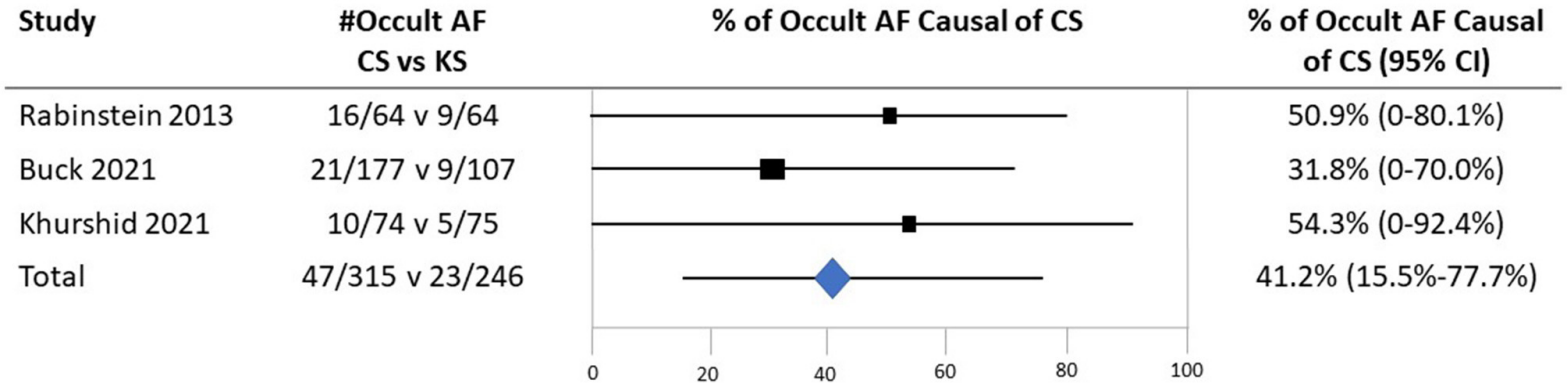
The probability that detected occult atrial fibrillation is causal in patients with CS based on case–control studies examining the prevalence of occult AF in cases with CS vs. controls with the stroke of a determined cause. Individual studies were identified by the first author and year of publication. The second column shows the prevalence of occult AF (# occult AF/total number of patients) in cases vs. controls. Black boxes with sizes corresponding to each study's weight in the analysis represent the point estimate of the probability that the PFO is causal with 95% CIs represented with the gray lines. The diamond in the last row represents the summary estimate of the probability.

A correction has been made to the Abstract Results paragraph.

This sentence previously stated:

“With the application of Bayes' theorem, the corresponding probabilities indicated that, when present, occult AF in patients with CS is causal in 38.2% (95% CI, 0–63.6%) of patients.”

The corrected sentence appears below:

“With the application of Bayes' theorem, the corresponding probabilities indicated that, when present, occult AF in patients with CS is causal in 41.2% (95% CI, 15.5–77.7%) of patients.”

A correction has been made to the Abstract Conclusion paragraph.

This sentence previously stated:

“Current evidence is preliminary, but it indicates that in cryptogenic stroke when occult AF is found, it is causal in about 38.2% of patients.”

The corrected sentence appears below:

“Current evidence is preliminary, but it indicates that in cryptogenic stroke when occult AF is found, it is causal in about 41.2% of patients.”

A correction has been made to Results, 5^th^ paragraph.

This sentence previously stated:

“The summary estimate derived from all three studies indicated that when present among patients with cryptogenic stroke, occult AF is causal in 38.2% (95% CI, 0–63.6%).”

The corrected sentence appears below:

“The summary estimate derived from all three studies indicated that when present among patients with cryptogenic stroke, occult AF is causal in 41.2% (95% CI, 15.5–77.7%).”

A correction has been made to Results, 5^th^ paragraph.

This sentence previously stated:

“Combining the rate of occult AF presence in patients with CS across all series (14.9%) and the frequency with which the detected occult AF is statistically deemed causal (38.2%) projects that occult AF accounts for 5.7% of all cryptogenic strokes (attributable risk).”

The corrected sentence appears below:

“Combining the rate of occult AF presence in patients with CS across all series (14.9%) and the frequency with which the detected occult AF is statistically deemed causal (41.2%) projects that occult AF accounts for 6.1% of all cryptogenic strokes (attributable risk).”

A correction has been made to Discussion, 1st paragraph.

This sentence previously stated:

“Findings indicated that when identified in patients with CS, occult AF is causally related to the index stroke in 38% and incidental in 62%.”

The corrected sentence appears below:

“Findings indicated that when identified in patients with CS, occult AF is causally related to the index stroke in 41% and incidental in 59%.”

A correction has been made to Discussion, 1st paragraph.

This sentence previously stated:

“Given the frequency with which occult AF is detected among patients with CS, the results suggest that occult AF is an important mechanism of CS, accounting for 1 in 17 of all cases of CS, including 1 in 16 cases in patients under the age of 65 years, and 1 in 18 cases in patients who are 65 years and older.”

The corrected sentence appears below:

“Given the frequency with which occult AF is detected among patients with CS, the results suggest that occult AF is an important mechanism of CS, accounting for 1 in 16 of all cases of CS, including 1 in 16 cases in patients under the age of 65 years, and 1 in 18 cases in patients who are 65 years and older.”

A correction has been made to Materials and Methods, subsection Probability of causal vs. incidental, paragraph 1.

This sentence previously stated:

“Probability occult AF is incidental in CS cases = (Prevalence of occult AF in controls) × (1 – Prevalence of occult AF in CS cases) (Prevalence of occult AF in CS cases) × (1 – Prevalence of occult AF in controls)”

The corrected sentence appears below:

“Probability occult AF is incidental in CS cases = [(Prevalence of occult AF in controls) × (1 – Prevalence of occult AF in CS cases)] ÷ [(Prevalence of occult AF in CS cases) × (1 – Prevalence of occult AF in controls)]”

The authors apologize for these errors and state that this does not change the scientific conclusions of the article in any way. The original article has been updated.

