# Supplementary material for: How often is occult atrial fibrillation in cryptogenic stroke causal vs. incidental? A meta-analysis
Source: Front Neurol. 2023 Mar 14;14:1103664. doi: 10.3389/fneur.2023.1103664 (PMC10043201; doi:10.3389/fneur.2023.1103664)
Supplement: Supplementary file 1 [file Table_1.DOCX]

**Supplement**

Table e1. Articles excluded after full-text review

| **Monitoring duration < 7 days** | |
| --- | --- |
| 1 | Arsava EM, Bas DF, Atalar E, Has AC, Oguz KK, Topcuoglu MA. Ischemic stroke phenotype in patients with nonsustained atrial fibrillation. Stroke 2015;46:634-40. |
| 2 | Sade LE, Keskin S, Can U, Çolak A, Yüce D, Çiftçi O, Özin B, Müderrisoğlu H. Left atrial mechanics for secondary prevention from embolic stroke of undetermined source. Eur Heart J Cardiovasc Imaging 2022;23:381-391. |
| 3 | Sposato LA, Klein FR, Jáuregui A, Ferrúa M, Klin P, Zamora R, Riccio PM, Rabinstein A. Newly diagnosed atrial fibrillation after acute ischemic stroke and transient ischemic attack: importance of immediate and prolonged continuous cardiac monitoring. J Stroke Cerebrovasc Dis 2012;21:210-6. |
| 4 | Yetim E, Topcuoglu MA, Canpolat U, Gocmen R, Oguz KK, Ozer N, Aytemir K, Arsava EM. Nonsustained Atrial Fibrillation in Ischemic Stroke Patients and Stroke-Free Controls From the Perspective of Stroke Pathophysiology. J Am Heart Assoc 2016;5:e004021. |
| **No known stroke cause comparator group** | |
| 5 | Arslan Y, Demirtaş BS, Ekmekci C, Tokuçoğlu F, Zorlu Y. The significance of Holter electrocardiography in the etiological evaluation of transient ischemic stroke. Brain Circ 2020;6:191-195. |
| 6 | Bielecka-Dabrowa A, Gasiorek P, Wittczak A, Sakowicz A, Bytyçi I, Banach M. Left Ventricular Diastolic Dysfunction as Predictor of Unfavorable Prognosis After ESUS. J Multidiscip Healthc 2021;14:617-627. |
| 7 | Noubiap JJ, Thomas G, Middeldorp ME, Fitzgerald JL, Harper C, Sanders P. Atrial fibrillation detection using insertable cardiac monitor after stroke: A real-world cohort study. J Cardiovasc Electrophysiol. 2023;34:142-146. |
| 8 | Scacciatella P, Jorfida M, Biava LM, Meynet I, Zema D, D'Ascenzo F, Pristipino C, Cerrato P, Giustetto C, Gaita F. Insertable cardiac monitor detection of silent atrial fibrillation in candidates for percutaneous patent foramen ovale closure. J Cardiovasc Med 2019;20:290-296. |

Table e2. Risk of Bias (assessed with ROBINS-I study tool)

|  |  | Buck et al | Khurshid et al | Rabinstein et al |
| --- | --- | --- | --- | --- |
| Selection Bias | Does the design or analysis control account for important confounding and modifying variables? | Definitely yes | Probably no | Definitely yes |
| Performance Bias | Did researchers rule out any impact from a concurrent intervention or an unintended exposure that might bias results? | Definitely yes | Probably yes | Probably yes |
| Attrition Bias | Did attrition result in a difference in group characteristics between baseline (or randomization) and follow-up? | Definitely no | Probably no | Definitely no |
| Detection bias | Were the outcome assessors blinded to the intervention or exposure status of participants? | Definitely no | Definitely no | Definitely no |
|  | Are the inclusion/exclusion criteria measured using valid and reliable measures, implemented consistently across all study participants? | Definitely yes | Definitely yes | Definitely yes |
|  | Are interventions/exposures assessed using valid and reliable measures, implemented consistently across all study participants? | Definitely yes | Probably no | Definitely yes |
|  | Are primary outcomes assessed using valid and reliable measures, implemented consistently across all study participants? | Definitely yes | Definitely yes | Definitely yes |
|  | Are confounding variables assessed using valid and reliable measures, implemented consistently across all study participants? | Definitely yes | Probably yes | Definitely yes |
| Reporting bias | Are the potential outcomes pre-specified by the researchers? Are all prespecified outcomes reported? | Definitely yes | Definitely yes | Definitely yes |
